# Survival from breast cancer in England and Wales up to 2001

**DOI:** 10.1038/sj.bjc.6604588

**Published:** 2008-09-23

**Authors:** A Leary, I E Smith

**Affiliations:** 1 Breast Unit, Royal Marsden Hospital, NHS Foundation Trust, Fulham Road, London SW3 6JJ, London, UK

Over the last 20 years, breast cancer has been something of a success story in the United Kingdom, with a 20% decrease in mortality despite a steadily increasing incidence. This is welcome news, but the underlying explanation is the topic of much debate. The widespread use of adjuvant medical therapies, the introduction of a national mammographic screening programme, and increased breast awareness all have their advocates. It is likely that all these factors are involved to a varying extent.

## The uncertain role of screening

The NHS breast cancer screening programme was introduced in 1988 after mortality from breast cancer had already begun to decrease and was based on the results from eight randomized trials showing that screening reduced mortality by 20–30% especially for women between 50 and 69 years of age. Since then, the methodology of these trials and the magnitude of survival benefit have been the subject of continuous debate (Olsen and Gotzsche, 2001; Baum, 2004). The absolute survival benefit from screening is difficult to quantify in the setting of concomitant advances in the management of breast cancer. Some critics argue that the harms of screening mammography, for example, false positives with resulting unnecessary procedures and increased anxiety, may actually outweigh the benefits (Gotzsche, 2004). What is certainly clear is that screening has led to a dramatic increase in the incidence of ductal carcinoma *in situ* (DCIS). DCIS is a non-invasive form of breast cancer, usually detected as asymptomatic microcalcifications on mammography, which may or may not progress to invasive cancer. Before the NHS screening programme, DCIS had been a rare diagnosis, but now it accounts for approximately 20% of screen-diagnosed breast cancers (Moss *et al*, 1995). When appropriately treated, this non-invasive cancer has an excellent prognosis (10-year RR of death from breast cancer, 2%; Ernster *et al*, 2000) and therefore has clearly contributed to the rising incidence of breast cancer while improving survival rates, but the impact on mortality of this dramatic increase in diagnosis remains uncertain. Reviews of autopsy series have revealed that DCIS is an incidental finding in 9% of women dying of non-breast cancer-related causes (Welch and Black, 1997), suggesting that screen-detected DCIS may lead to overdiagnosis in some women. Likewise, retrospective studies have demonstrated that only 15–50% of untreated DCIS will progress to invasive disease (Erbas *et al*, 2006). The problem lies in identifying which ones will.

## Breast awareness

Despite the high media profile for breast self-examination (BSE), an approach of systematic BSE has not been shown to improve survival and may actually cause anxiety and unnecessary biopsies (Kosters and Gotzsche, 2003). Breast awareness, however, is still promoted and involves encouraging women to recognise and report without delay any unusual breast changes (McCready *et al*, 2005). Improved public education about breast cancer combined with increased screening has led to a stage shift, with more women presenting with early disease.

## Advances in the treatment of early breast cancer

### Loco-regional treatment

Surgery has always been central to the management of early breast cancer. Over the last few decades, however, it has become clear that access to surgical specialists is an important predictor of long-term outcome. Women with early breast cancer treated by specialist surgeons have lower local recurrence rates and an improved survival compared with those treated by non-specialists (Kingsmore *et al*, 2004). This benefit is in part attributable to higher rates of adequate local treatment of the breast and/or axilla. Increased access to specialist surgeons and the establishment of national guidelines for the surgical management of breast cancer (Quality assurance guidelines for surgeons in breast cancer screening, in Publication No. 20, N. BSP, Editor, 1992) have therefore contributed to survival improvements. Similarly, standardisation of pathology reporting and the use of specialized breast pathologists have been important factors in improving the quality of care delivered to women with breast cancer (Program, UNS, *Pathology Reporting in Breast Cancer Screening*, in *2nd edition*, NPN 3, Editor, 1997: Sheffield UK). Finally, radiotherapy after breast-conserving surgery and after mastectomy in selected high-risk patients has been shown to improve not only loco-regional control, but also long-term survival (5% reduction in 15-year breast cancer mortality) (Clarke *et al*, 2005).

### Systemic treatment

The last two decades have seen tremendous advances in the adjuvant treatment of early breast cancer. In 1985, the Nolvadex Adjuvant Trial Organisation study was the first randomised trial to show an overall survival advantage in patients receiving adjuvant treatment with the selective oestrogen receptor modulator, tamoxifen (NATO, 1985). Since then, a series of reviews have confirmed that 5 years of post-operative tamoxifen for pre- and post-menopausal women with ER+ breast cancer reduces mortality by over 25% at up to 15 years of follow-up (EBCTCG, 1998a; EBCTCG, 2005).

Adjuvant chemotherapy trial data also began to emerge in the early 1980s, again suggesting a survival benefit particularly for young women (EBCTCG, 1988). Subsequent overview results confirmed a proportional mortality reduction of 27 and 11% among women below and above the age of 50, respectively, regardless of nodal status (EBCTCG, 1998b). Efforts have been made to determine the optimal cytotoxic regimen. The standard post-operative treatment in most early trials was CMF polychemotherapy (cyclophosphamide, methotrexate, and 5-fluorouracil), but recent evidence suggests that there is a small but highly significant further survival benefit with anthracycline-based regimens (RR=0.89; 2 *P*<0.00001) (EBCTCG, 2005), and these have become widely accepted as standard adjuvant chemotherapy.

The most recent overview suggests that breast cancer mortality may actually be halved by a combination of endocrine therapy and chemotherapy after surgery in women with hormone receptor-positive disease (EBCTCG, 2005). As these account for more than two-thirds of newly diagnosed breast cancer patients, it is likely that the increasing use of adjuvant therapy has had an important function in breast cancer survival improvement.

## Advances in the treatment of metastatic breast cancer

A number of new cytotoxic, endocrine and targeted agents have been licensed for breast cancer over the last 15 years and have greatly increased treatment options for advanced disease (Smith, 2002). Although the impact on overall survival trends is likely to be modest, the incorporation of these new therapies into clinical practice has been shown to significantly prolong survival from metastatic breast cancer (OS from metastatic breast cancer: 438 days *vs* 667 days for women treated in 1991–1992 *vs* 1999–2001; Chia *et al*, 2007). By increasing the number of effective anti-cancer agents, and improving palliative strategies by offering radiotherapy for symptomatic bone and brain lesions, or surgical treatment of spinal and brain metastases, tremendous gains have been achieved in improving both the quality of life and survival of women with advanced breast cancer.

## The deprivation gap

Quinn *et al* (2008) draw attention to the observation that although the incidence of breast cancer has risen more rapidly among affluent women, the deprivation gap (difference in survival from breast cancer for women from affluent *vs* deprived background) has not changed. This may in part be due to the fact that women from a higher socioeconomic background are more likely to undergo mammography and therefore be diagnosed with a small screen-detected breast cancer (Taylor and Cheng, 2003). However, as Quinn *et al* (2008) point out, if screening were the sole reason for the increased incidence, this should have led to a stage shift in the affluent group and a widening in the deprivation gap. Women of higher socioeconomic status are also at increased risk of developing invasive breast cancer due to differing reproductive behaviours of aetiological significance, for example, late age at first birth, low parity, and low breastfeeding rates (Kelsey *et al*, 1981). The incidence trend may therefore also reflect changes in reproductive habits and possibly in the use of hormone replacement therapy among affluent women.

## Optimism for the future

On the premise that adjuvant medical therapies are a major factor in survival improvement, it is likely that this trend will continue with the emergence of new and more effective therapies.

Several trials have shown that adjuvant aromatase inhibitors achieve superior disease-free survival to tamoxifen in post-menopausal women, and it seems likely that this will translate into a further small survival improvement (Lin and Winer, 2008). Specifically, the MA-17 trial has demonstrated a significant improvement in disease-free survival for letrozole *vs* placebo after 5 years of tamoxifen and a significant overall survival benefit in women with node-positive disease (HR=0.61, *P*=0.04) (Goss *et al*, 2005), raising the concept of long-term maintenance endocrine therapy for 10 years or more in selected patients.

Adjuvant trastuzumab (Herceptin) has recently shown an impressive 50% reduction in the risk of early recurrence and a 30% reduction in mortality relative to placebo in women with HER2-positive breast cancer (Romond *et al*, 2005; Dinh *et al*, 2007). Although this involves only 15% of breast cancer patients, these represent a high-risk group, and the proof of principle gives optimism for further targeted therapies now in development.

Perhaps as important as the development of novel therapies is the development of tools to identify women with good prognosis who could benefit from more conservative treatment. Some of the new techniques include sentinel node biopsy to identify patients who may be spared axillary dissection and gene expression profiles to identify women at low risk of recurrence who may be spared adjuvant chemotherapy.

Finally, the changes in cancer treatment provision such as the increasing use of multidisciplinary teams, one-stop triple assessment clinics for symptomatic breast complaints and breast cancer nurse specialists will undoubtedly continue to optimise the quality of care offered to women with breast cancer.

## Conclusion

Improvements in breast cancer diagnosis and treatment in the UK have had an important impact on survival, and it is reasonable to expect that the outlook will continue to improve over the next decade. Governmental guidelines implemented in the late 1990s designed to reduce delays and improve multidisciplinary organisation and the continuing development of more effective medical therapies are both making important contributions. The political challenge is to ensure equal access to best care for women from deprived backgrounds. The medical challenge is to select through molecular profiling which patients are most likely to benefit from the ever-expanding medical treatment options and which can be spared unnecessary toxicities. The good news is likely to continue.
